# Skeletal and body composition effects of orchiectomy in a colony of diet-induced obese cynomolgus macaques

**DOI:** 10.1093/jbmrpl/ziag150

**Published:** 2026-09-01

**Authors:** Melissa A Kirigiti, Lindsay Bader, Cicely Zaro, Casey M McGuire, Jesper J Hansen, Berit O Christoffersen, Ellen M Straarup, Paul Kievit, Kristin A Sauter

**Affiliations:** Division of Metabolic Health and Disease, Oregon National Primate Research Center, Oregon Health and Science University, Beaverton, OR 97006, United States; Division of Metabolic Health and Disease, Oregon National Primate Research Center, Oregon Health and Science University, Beaverton, OR 97006, United States; Division of Metabolic Health and Disease, Oregon National Primate Research Center, Oregon Health and Science University, Beaverton, OR 97006, United States; Division of Metabolic Health and Disease, Oregon National Primate Research Center, Oregon Health and Science University, Beaverton, OR 97006, United States; Obesity Pharmacology, Obesity Novo Nordisk A/S, Måløv, Denmark; Obesity Pharmacology, Obesity Novo Nordisk A/S, Måløv, Denmark; Obesity Pharmacology, Obesity Novo Nordisk A/S, Måløv, Denmark; Division of Metabolic Health and Disease, Oregon National Primate Research Center, Oregon Health and Science University, Beaverton, OR 97006, United States; Division of Metabolic Health and Disease, Oregon National Primate Research Center, Oregon Health and Science University, Beaverton, OR 97006, United States

**Keywords:** hypogonadism, osteoporosis, sarcopenia, cynomolgus macaques, obesity

## Abstract

Obesity is a leading cause of secondary hypogonadism or low testosterone. Conversely, hypogonadism frequently leads to excess body fat/obesity demonstrating a potentially reciprocal relationship between the two. This study describes the impact of bilateral orchiectomy (ORX) in a colony of diet-induced obese (DIO) male Mauritius cynomolgus macaques (DIO-CM) maintained on a Western Style Diet (WSD). Body composition analysis via DXA was performed prior and up to 15 m postsurgery to determine the long-term changes in hypogonadism. As expected, ORX significantly reduced testosterone, inhibin B, and estrogen while increasing follicle-stimulating hormone and luteinizing hormone throughout the entire follow-up period. Animals lost significant body weight mainly due to a loss of lean but not fat mass. Orchiectomy had detrimental and long-lasting, 15 m postsurgery, effects on bone, including decreased total BMC and BMD, with the most drastic losses localized to the pelvic region. Additionally, animals demonstrated a transient elevation of numerous bone turnover markers. This model demonstrates that in a phenotypically well-characterized colony of DIO-CM, hypogonadism induces lean mass loss, increased body fat percentage, and numerous hallmarks of osteoporosis. This nonhuman primate model is relevant to human bone biology, age-related body composition changes, such as sarcopenic obesity, and hypogonadism and therefore could be utilized to test muscle and bone mass preserving interventions in the future.

## Graphical abstract

**Graphical Abstract:**
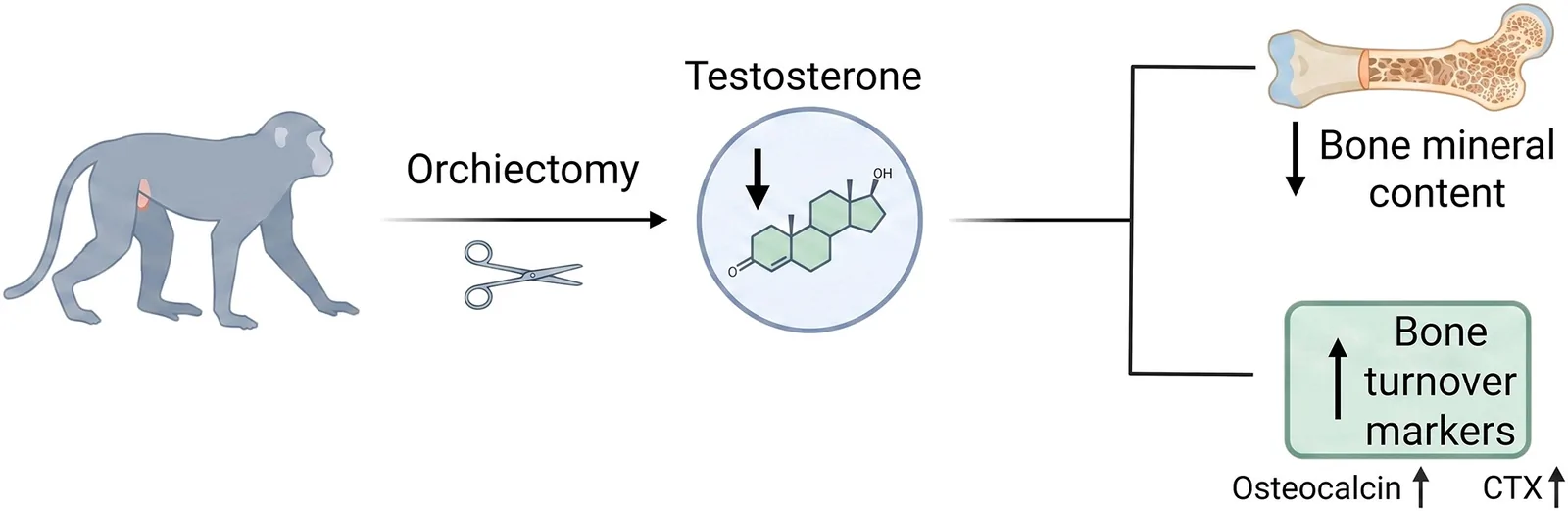

Graphical abstract was created in BioRender. Sauter, K. (2026) https://BioRender.com/1ugnvip

## Introduction

Sex steroids, such as androgens and estrogens, are essential for skeletal growth/maturation to develop peak bone mass, as well as in older age for maintaining bone and skeletal muscle mass and health. Osteoporosis, loss of muscle mass and changes in fat distribution in aging is driven by decreases in sex steroids, most clearly demonstrated by menopause-associated loss of estrogen leading to osteoporosis in women. Conversely, the cause of osteoporosis in elderly men is less straightforward; however, age-associated loss of testosterone, and therefore less substrate available for conversion into estrogen, may be a contributing factor. While osteoporosis is less common in men than women, roughly one-third of hip fractures happen in men, although fracture incidence is delayed by at least a decade compared to women.^1^ In addition to the age-associated loss of testosterone, androgen deprivation therapy (ADT) is another reason for low levels of sex steroids in males. Androgen deprivation therapy is standard of care for males with some forms of prostate or testicular cancer and can produce hypogonadism either via bilateral surgical removal of the testes (orchiectomy/ORX) or chemical castration with a gonadotropin-releasing hormone agonist.^2^ Bone and muscle health post-ADT treatment has become the subject of clinical research focused on sarcopenia, osteoporosis, and epidemiological studies on the risk of fractures.^3–5^ However, patients receiving ADT often have complicated preexisting health conditions prior to castration making it difficult to ascertain what castration-specific bone changes occur longitudinally. A small study examined 12 young adult males who underwent ORX due to sexual delinquency and demonstrated bone loss compared to age and sex matched controls.^6^ However, the studies were not longitudinal and on average started years after the ORX surgery.

Obesity prevalence is higher among people with cancer and is a risk factor for the development of many different types of cancer. Obesity is associated with poor prostate cancer prognosis and can impact treatment effectiveness.^7^ Studies document that nearly 43% of men were overweight at prostate cancer diagnosis.^8^ Additionally, non-obese men are at high risk of becoming obese due to treatment side effects.

Animal models of sex steroid loss are essential for the study of hypogonadal sarcopenia and osteoporosis, both for investigating the molecular underpinnings of muscle and bone loss as well as to develop novel therapies. The Food and Drug Administration (FDA) recommends utilizing the ovariectomized (OVX) rat and a second non-rodent model for postmenopausal osteoporosis therapy research.^9^ For males, an ORX-model in rat has been proposed to simulate male osteoporosis due to hypogonadism.^10^^,^^11^ However, an important complication to studying bone in the rodent skeleton is that, unlike humans, rodents maintain a growth plate into old age with a potential for longitudinal bone growth late in their lifespan^12^ introducing a variable that is not present in humans. Nonhuman primates (NHP), similar to humans, experience mineralization of their growth plates and replicate important aspects of human physiology.^13^^,^^14^ Additionally, NHP experience a similar aging process to humans and are utilized for aging research,^15^ as a translational model of menopause^16^ and to study bone loss.^17^ Postmortem bone analysis of castrated NHP males showed osteoporosis of vertebrae and femur as well as thinning of the skull,^18^ thus demonstrating that the NHP indeed could be a valuable model to further investigate the role of sex steroids in maintaining bone. In addition, NHP, particularly rhesus macaques (*Macaca mulatta*), are established translational models of age-related sarcopenia, exhibiting progressive skeletal muscle mass loss, preferential type II fiber atrophy, and increasing mitochondrial dysfunction across aging in long-term longitudinal studies.^19^^,^^20^ In contrast, the effects of castration on skeletal muscle mass and sarcopenia-like changes are less well explored.

In this study, we examined the changes in body composition and bone-related parameters in a colony of male Mauritian diet-induced obese cynomolgus macaques (DIO-CM), to more closely match the population of overweight men at prostate cancer diagnosis, before and after ORX. Using DXA, we document the changes in lean, fat, and bone mass up to 15 m after ORX, including accompanying circulating hormones.

## Materials and methods

### Animal care

All animal procedures were conducted in accordance with the guidelines of the Institutional Animal Care and Use Committee of the Oregon National Primate Research Center (ONPRC) and Oregon Health and Science University. The ONPRC abides by the Animal Welfare Act and Regulations enforced by the USDA, the Public Health Service Policy on Humane Care and Use of Laboratory Animals, in accordance with the Guide for the Care and Use of Laboratory Animals of the National Institutes of Health.

Male Mauritius cynomolgus macaques were purpose bred at BioCulture US LLC and group housed according to the Novo Nordisk Nonhuman Primate Behavioral Management Plan at the ONPRC in custom designed pens (Carter2 Systems, Inc.). The pens were designed to house social groups of animals, ranging from pair-housed to larger groups. The pens contained shelving, climbing structures, verandas, and perches with thermo-neutral solid surfaces and all pens had solid flooring with alternating weeks of bedding or pools for enrichment. Animals were housed in a 12/12 d/night light cycle, with lights gradually turning on or off over a 30-min period to simulate sunrise and sunset. The dimensions of each pen were 2 m wide × 1.3 m deep × 2.6 m high with a maximum of 3 animals per pen; larger groups had access to multiple pens.

Male Mauritius cynomolgus macaques were maintained on a commercially available Western Style Diet (WSD) (36% calories from fat vs 14.6% in typical control diet, 18.2% protein and 46% from carbohydrates, Purina Mills Inc.; 3.52 kcal/g of metabolizable energy) for more than 4 yr prior to study start. Animals were in a steady state regarding weight and body fat percentage prior to study start. Diets are sufficient in vitamin, mineral, and protein content for normal growth.

### Bilateral orchiectomy

**Figure 1:**
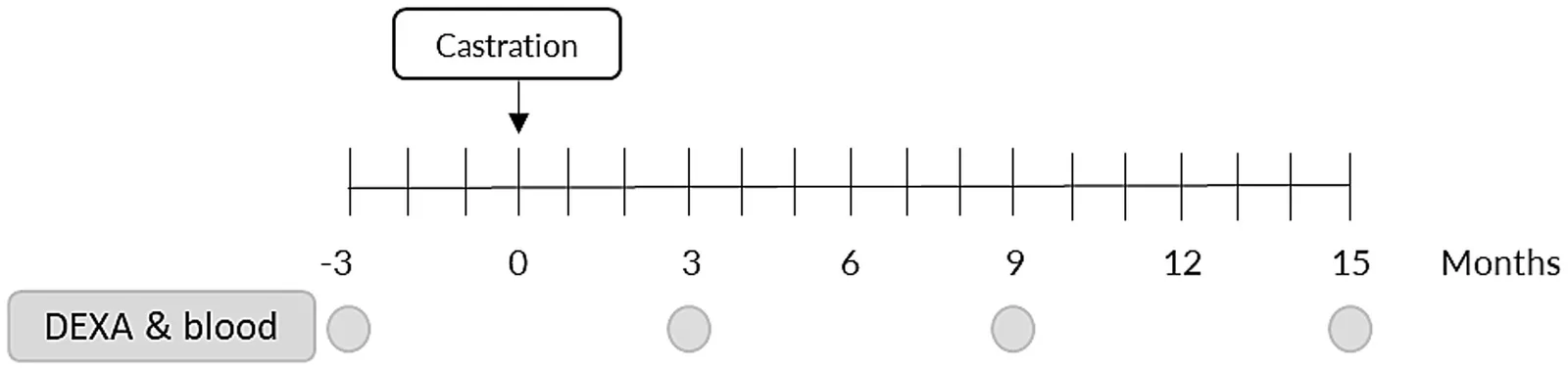
Experimental timeline.

Twenty young adult males from a colony of DIO-CM underwent bilateral orchiectomy (ORX) according to the timeline shown in Figure 1. A ventral midline skin incision was made cranial to the scrotum, with blunt dissection to reveal the testicular tunic. An incision was made through the tunic and the testis were delivered manually. The spermatic cord (vas deferens, cremaster m., and testicular vasculature) were clamped then double-ligated (one circumferential, one transfixing ligature) with 3-0 Vicryl, transected, and the testis were removed. The contralateral testis were resected in like manner.

### Dual-energy X-ray absorptiometry

Determination of total, lean, and fat mass, as well as BMC and BMD were assessed using DXA scanning (Lunar iDXA, GE HealthCare). After an overnight fast, animals were sedated with 3-5 mg/kg Telazol, weighed, underwent blood collection, and were placed in a prone position during a standard total body scan. We utilized ROI to examine localized areas in subsequent analysis.

### Blood

In conjunction with DXA scanning, animals were fasted overnight and underwent blood collection. Hematology: complete blood cell counts (CBC) were performed by the ONPRC Clinical Pathology department on an ABX PENTRA 60C+ hematology analyzer. Clinical chemistry panel was determined using an ABX Pentra 400 chemistry analyzer (Horiba Diagnostics) by the ONPRC Clinical Pathology department. HbA1c was measured using a Siemens DCA Advantage unit. Blood glucose was measured immediately using a hand-held glucometer and a separate sample was analyzed on the EKF Glucose analyzer. Insulin was measured in circulation using a commercial ELISA assay according to the manufacturer’s instructions (Alpco, 80-INSHU-E01.1). HOMA-IR was calculated as (fasting glucose X fasting insulin)/405.

Blood was aliquoted as plasma or serum and frozen at −80 to be assayed at a later date. Testosterone, estrogen, follicle-stimulating hormone (FSH), luteinizing hormone (LH), C-terminal telopeptide of type I collagen (CTX), osteocalcin, total 25OHD, and hair cortisol were assayed on a Roche cobas e411 automated clinical platform; Inhibin B, PTH and adiponectin (ALPCO total adiponectin cat# 80-ADPHU-E01) were assayed via ELISA; Leptin was assayed via RIA (Millipore cat# HL-81K), all by the ONPRC Endocrine Technologies Core.

### Statistical analysis

Statistical analyses were performed using Prism software. Significance was calculated using repeated measures one-way ANOVA with Tukey’s multiple comparisons test. Correlations were determined using Pearson’s correlation coefficient. A *p*-value <.05 was considered significant.

## Results

Twenty young adult males, from a colony of DIO-CM maintained on WSD (Table 1) underwent bilateral orchiectomy (ORX) according to the timeline shown in Figure 1. As published by our group previously, macaques maintained on WSD have varying responses and naturally segregate into a larger diet-sensitive group, becoming obese and insulin resistant, and a smaller diet-resistant group.^21^ This colony demonstrates this phenomenon specifically in the range of BW, body fat %, and FBI **(**Table 1**)**. However, starting body fat % did not correlate with change in bone or bone markers post-ORX (data not shown). Epiphyseal fusion occurs on average at 6.5 yr of age in male CM.^22^^,^^23^ Therefore, at time of ORX these 7-yr-old males are expected to have undergone fusion.

**Table 1:** Baseline demographics of a colony of DIO-CM.

*N*	20
**Age (yr)**	6.65 ± 0.11 (6-7)
**BW (kg)**	10.3 ± 0.35 (7.7-13.75)
**Body fat (%)**	27.9 ± 2.15 (16.3-49.4)
**FBG (mg/dL)**	55.9 ± 2.39 (34-87)
**FBI (**μ**U/mL)**	21.6 ± 3.15 (4.27-52.12)
**HOMA-IR**	3.69 ± 0.69 (0.54-12.57)
**HbA1C (%)**	4.23 ± 0.06 (3.8-5)
**CHOL (mg/dL)**	121.4 ± 5.79 (80-175)
**TRIG (mg/dL)**	40.4 ± 4.98 (21-105)
**HDL (mg/dL)**	67.2 ± 3.01 (47-103)
**LDL (mg/dL)**	42.4 ± 3.26 (19-72)

Values at baseline are shown for age, body weight (BW), body fat (%), fasting glucose (FBG), fasting insulin (FBI), HOMA-IR, HbA1c, cholesterol (CHOL), triglycerides (TRIG), HDL, and LDL. Data are expressed as mean ± SEM (range).

**Figure 2:**
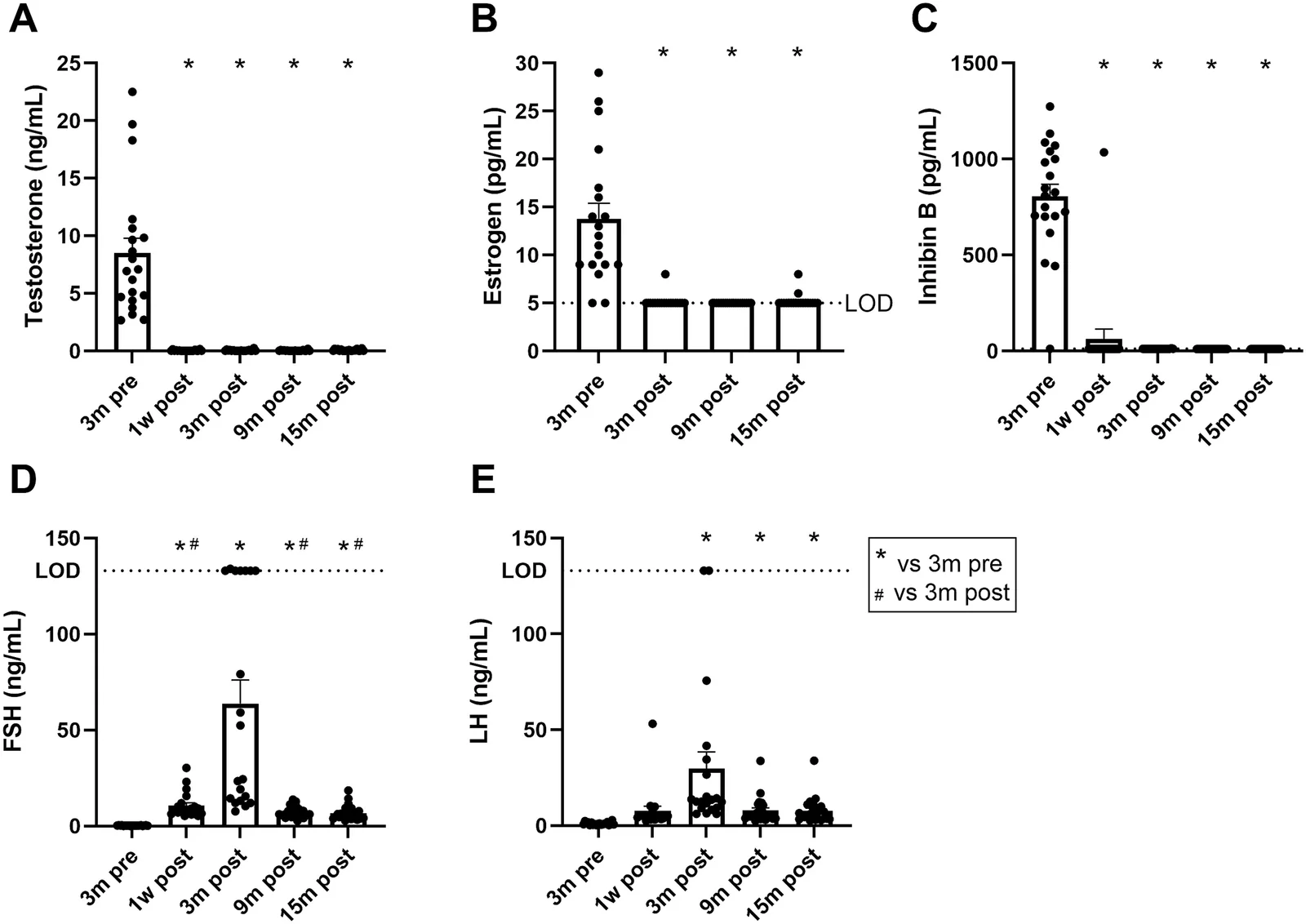
Orchiectomy-induced changes in circulating hormone values. Fasted circulating levels of the hormones: testosterone (A), estrogen (B), inhibin B (C), follicle-stimulating hormone (FSH) (D), and luteinizing hormone (LH) (E) were measured at baseline and up to 15 m post-orchiectomy. Data are expressed as mean ± SEM. ^*^p < .05 by repeated measures one-way ANOVA with Tukey’s multiple comparisons test.

As expected, removal of the gonads caused circulating testosterone, estrogen and inhibin B to significantly decrease (*p* < .0001) as rapidly as 1-wk post-ORX and levels of these three hormones remained nearly undetectable up to the last time point 15 m postsurgery (Figure 2A-C). Average testosterone levels at baseline were 8.52 *±* 1.28 ng/mL and at 15 m post-ORX the group average was 0.08 *±* 0.02 ng/mL with more than half of the animals below the detectable limit of 0.025 ng/mL. The DIO-CM had a large spread of baseline testosterone values (2.68-22.50 ng/mL) as well as baseline estrogen values (<5-29 pg/mL) but interestingly neither of these values correlated with baseline BMC or lean mass (data not shown). However, baseline testosterone tended to positively correlate with baseline estrogen (*p* = .0567, data not shown). With diminished levels of testosterone and inhibin B, the negative feedback loop was stunted and circulating levels of FSH (*p* < .0001) and LH increased by 1 wk post-ORX, peaked at 3 m post (FSH 63.77 *±* 1.28 ng/mL and LH 29.82 *±* 8.70 ng/mL), and remained statistically significantly elevated (FSH more than 20×, *p* < .0001 and LH more than 7×, *p* = .0015) above baseline levels up to 15 m after ORX (Figure 2D and E). Interestingly, at 3 m, there were a few animals that had levels above the assay detection limits for both FSH and LH.

**Figure 3:**
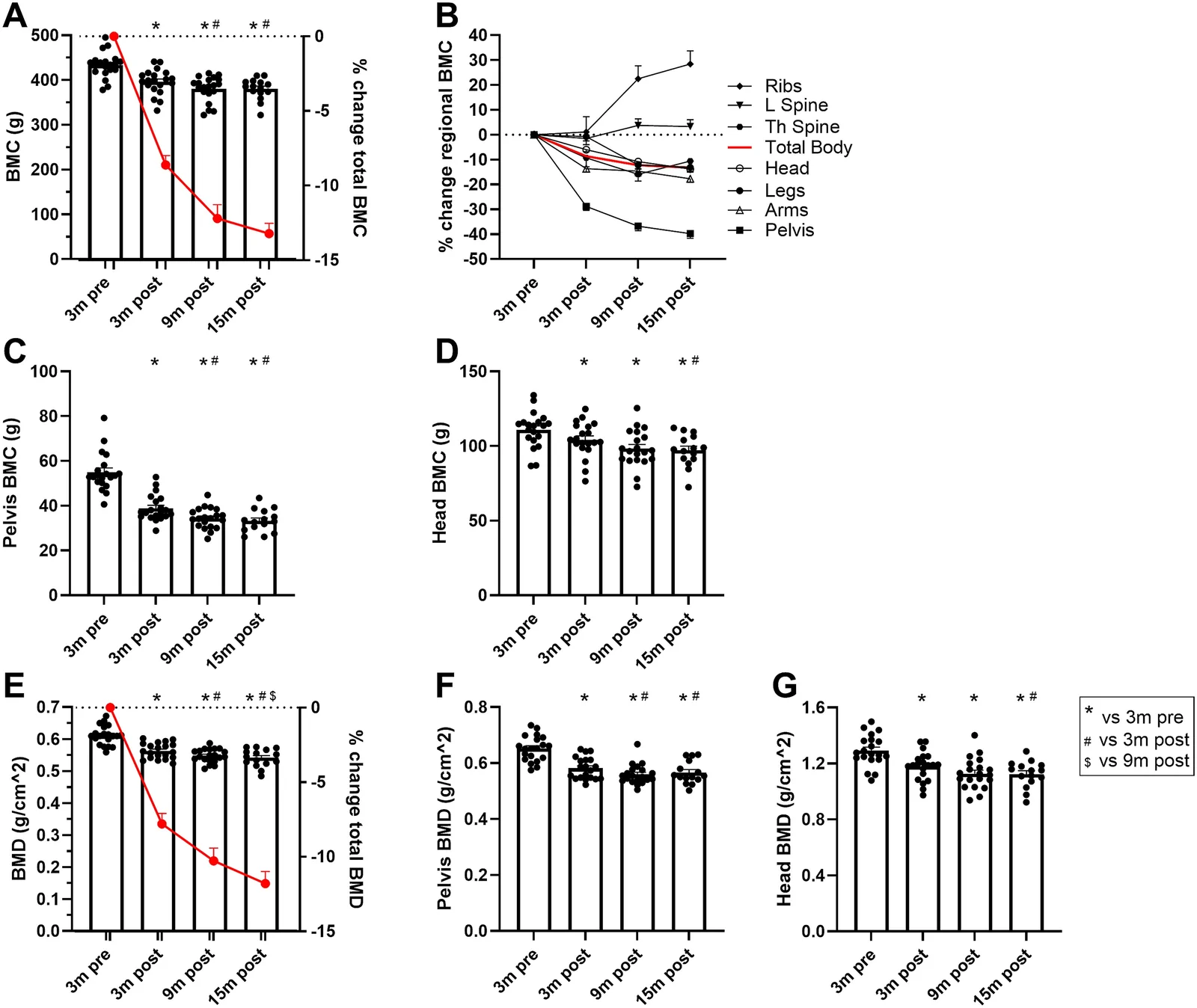
Orchiectomy-induced decreases in BMC and density. Change in BMC in grams [left axis] and %change BMC vs baseline (right axis) (A), % change in regional BMC (B), pelvic BMC (C), head BMC (D), change in BMD in g/cm^2^ [left axis] and %change BMD vs baseline (right axis) (E), pelvic BMD (F), head BMD (G). ^*^p < .05 by repeated measures one-way ANOVA with Tukey’s multiple comparisons test.

The DIO-CM had significant bone loss post-ORX measured in BMC as well as loss of BMD. Total body BMC dropped by more than 50 g post-ORX (3 m pre was 433.6 *±* 6.3 g vs 15 m post was 380.2 *±* 6.1 g, *p* < .0001, Figure 3A). However, the greatest decline in total body BMC occurred rapidly within the first 3 m postsurgery (3 m post: −8.6 ± 0.6%, Figure 3A). Each body region was analyzed for BMC loss compared to total body BMC loss. The region with the largest disproportionate BMC loss was pelvis (−39.77%) while thoracic (Th) spine, head, legs, and arms (between −10% to −18%) matched the total body BMC loss of 13%. Only chest cavity (ribs/clavicle/scapula, not vertebrae) and Lumbar (L) Spine were protected without BMC loss (Figure 3B). The pelvic BMC loss accounted for more than 20 g out of the 50 g of total body loss (3 m pre was 54.9 *±* 1.9 g vs 15 m post was 33.3 *±* 1.3 g, Figure 3C). Additionally, the head accounted for a BMC loss of more than 13 g out of the 50 g of total body loss (3 m pre was 110.9 *±* 2.7 g vs 15 m post was 97.2 *±* 2.8 g, Figure 3D). The remaining total body BMC loss was divided among extremities with little change in spine BMC (data not shown). Total body BMD also significantly decreased; pre-ORX BMD was 0.612 *±* 0.007 g/cm^2^ and 15 m postsurgery it dropped to 0.541 *±* 0.007 g/cm^2^  *p* < .0001, (Figure 3E). Again, the greatest decline in total body BMD occurred rapidly within the first 3 m postsurgery (3 m post: −7.8 ± 0.7%, Figure 3E). These losses were region specific and most drastic when examining both pelvis and head (Figure 3F and G).

**Figure 4:**
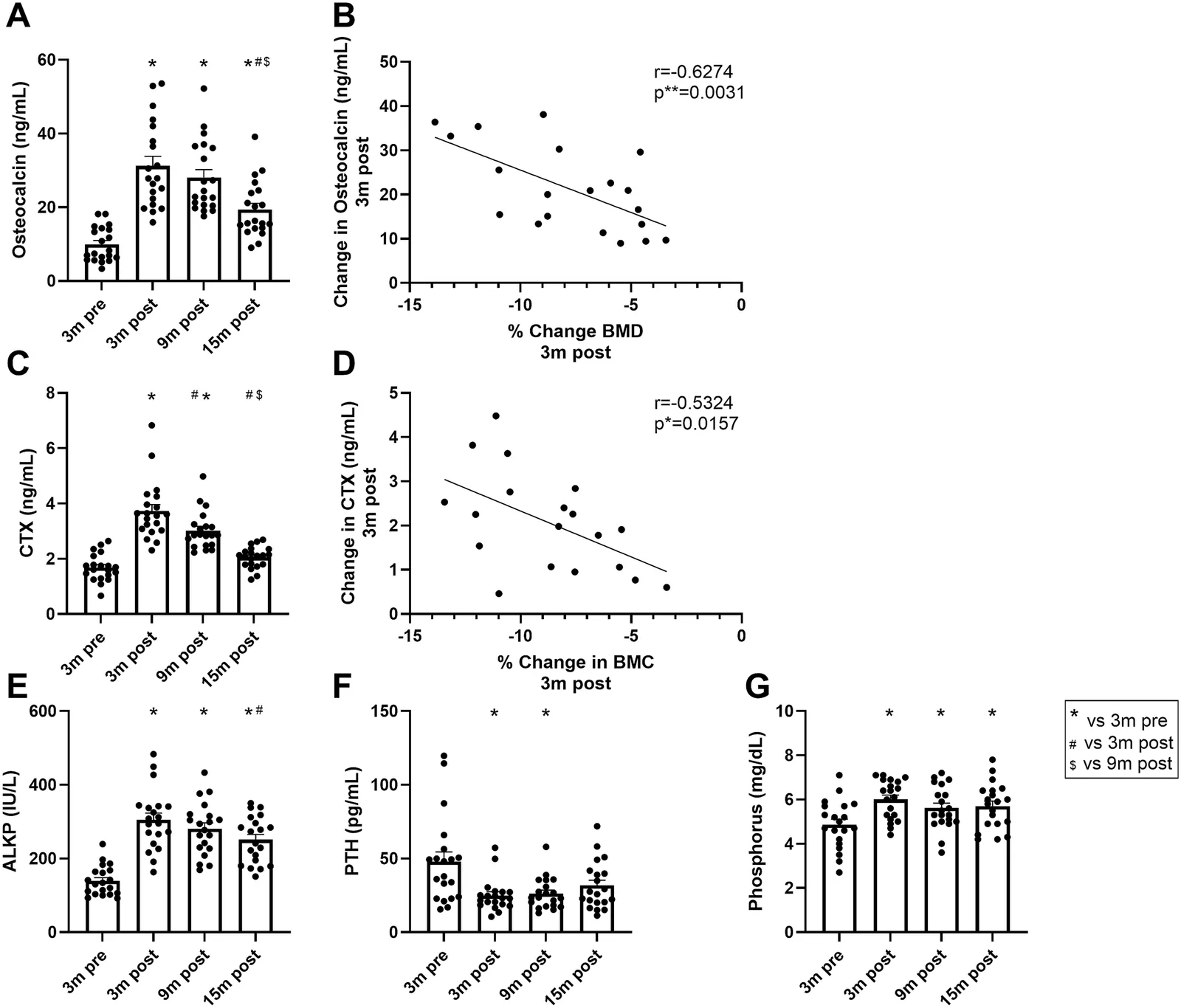
Orchiectomy-induced changes in circulating markers of bone health. Fasted circulating levels: osteocalcin (A), C-terminal telopeptide of type I collagen (CTX) (C), alkaline phosphatase (ALKP) (E), PTH (F), and phosphorus (G) were measured at baseline and up to 15 m post-orchiectomy. Data are expressed as mean ± SEM. ^*^p < .05 by repeated measures one-way ANOVA with Tukey’s multiple comparisons test. Correlations were determined by Pearson’s correlation coefficient between: 3 m change in osteocalcin vs baseline and 3 m % change in BMD vs baseline (B) and 3 m change in CTX vs baseline and 3 m % change in BMC vs baseline (D).

We also examined circulating bone turnover markers (BTMs) as well as enzymes and minerals indicative of bone health. Osteocalcin is a marker of bone formation and was significantly elevated with peak values at 3 m post-ORX (baseline: 9.92 *±* 1.04 ng/mL vs 3 m: 31.23 *±* 2.30 ng/mL, *p* < .0001, Figure 4A). The increase in osteocalcin at 3 m correlated with percent decrease in total body BMD at 3 m post-ORX, *p* = .0031 (Figure 4B). At 15 m osteocalcin remained significantly elevated (*p* < .0001), roughly 2× higher than baseline. C-terminal telopeptide of type I collagen is a marker of bone resorption and was also significantly elevated with peak values at 3 m post-ORX (baseline: 1.69 *±* 0.11 ng/mL vs 3 m: 3.73 *±* 0.23 ng/mL, *p* < .0001, Figure 4C). The increase in CTX at three months correlated with percent decrease in total body BMC at 3 m post-ORX, *p* = .0157 (Figure 4D). C-terminal telopeptide of type I collagen decreased faster than osteocalcin and by 15 m there was no significant difference from baseline (*p* = .0598). Elevated circulating levels of both BTMs indicate increased bone turnover. Alkaline phosphatase (ALKP) is an enzyme involved in bone turnover and it was also significantly elevated post-ORX and remained higher even 15 m post, *p* < .0001, (Figure 4E). Animals also demonstrated a significant decrease in PTH postsurgery, 3 m post *p* = .0024 and 9 m post *p* = .0063 (Figure 4F), which lead to increased circulating phosphorus, 3 m *p* < .0001 (Figure 4G). There was no change in circulating magnesium or vitamin D levels (data not shown). At baseline, none of these circulating markers of bone health correlated to the baseline BMC (data not shown).

**Figure 5:**
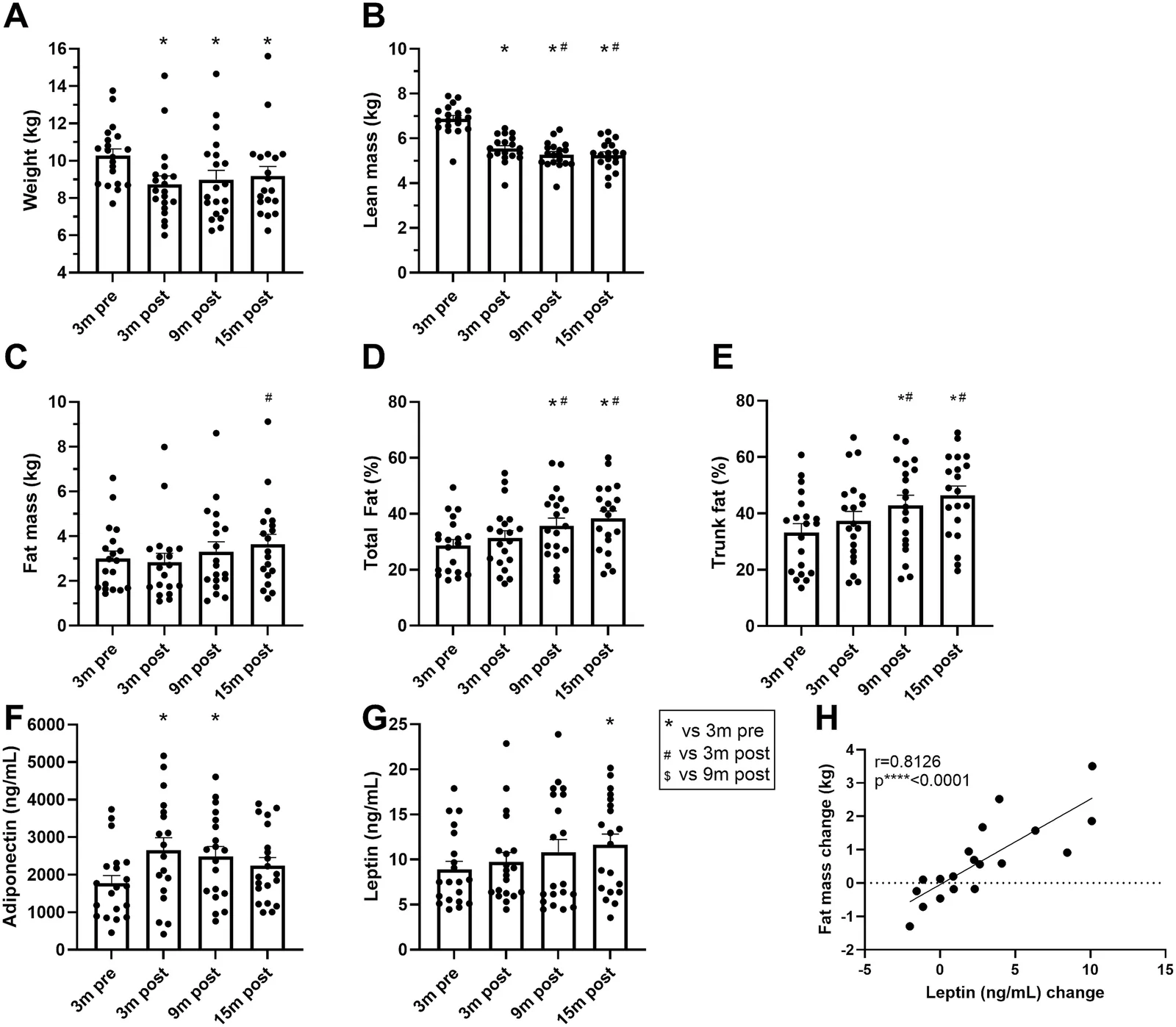
Orchiectomy-induced changes in body composition and adipokines. Body weight (kg) (A), lean mass (kg) (B), fat mass (kg) (C), total fat (%) (D), trunk fat (%) (E), fasted adiponectin (F), and fasted leptin (G) were measured at baseline and up to 15 m post-orchiectomy. Data are expressed as mean ± SEM. ^*^p < .05 by repeated measures one-way ANOVA with Tukey’s multiple comparisons test. Correlation was determined by Pearson’s correlation coefficient between 15 m fat mass change (kg) vs baseline and 15 m leptin (ng/mL) change vs baseline (H).

DIO-CM, similar to mice,^24^ but not domestic pets,^25^^,^^26^ minipigs,^27^ and humans,^28^^,^^29^ lost significant body weight post-ORX (Figure 5A). During initial baseline body composition assessments 3 mo prior to surgery their average body weight was 10.28 *±* 0.36 kg. Follow-up weights 3 m postsurgery demonstrated that animals had lost significant weight (average body weight dropped by more than 1.5 kg to 8.73 *±* 0.45 kg, *p* < .0001). Body composition via DXA demonstrated that the weight loss was mainly due to a significant loss of lean mass and not fat mass. Lean mass significantly decreased by more than 1 kg at 3 m post-ORX (3 m pre: 6.87 *±* 0.15 kg vs 3 m post: 5.54 *±* 0.14 kg, *p* < .0001) and declined even further 9 m post (Figure 5B). The animals remained fairly consistent in total fat mass prior to and postsurgery (Figure 5C). However, due to the animals’ lean mass loss with stable fat mass, the total body fat percentage significantly increased by 9 m (*p* = .0023) and peaked at 15 m post-ORX (3 m pre: 28.7 *±* 2.2% vs 15 m post: 38.3 *±* 2.7%, *p* = .0003; Figure 5D). In addition, when region specific trunk fat was analyzed, the animals tended to increase as quickly as 3 m postsurgery reaching significant increases by 9 m, *p* = .0015 (Figure 5E). Animals also demonstrated an initial significant increase in circulating adiponectin postsurgery (3 m post *p* = .0148) with the corresponding drop in body weight. Circulating leptin also significantly increased but not until 15 m postsurgery (*p* = .0154), where the animals were weight stable and had a trend towards increased fat mass (Figure 5F and G). The increase in circulating leptin levels (15 m post-ORX vs BL) strongly correlated with increase in total fat mass over the same time period (*p* < .0001, Figure 5H).

**Figure 6:**
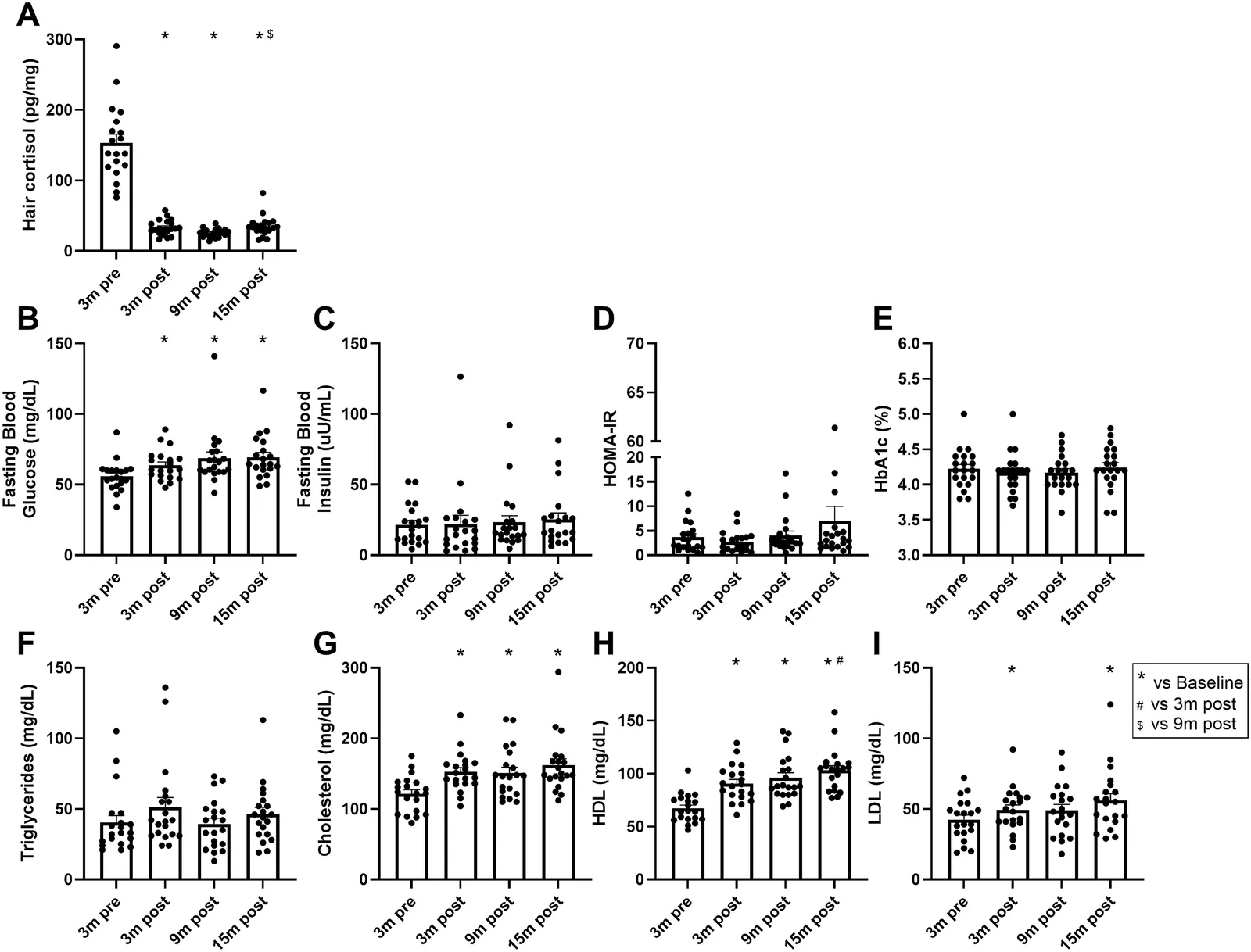
Orchiectomy-induced changes in hair cortisol, circulating measures of glucose homeostasis and blood lipids. Hair cortisol (pg/mg) (A), fasting blood glucose (mg/dL) (B), fasting blood insulin (μU/mL) (C), HbA1c (E), triglycerides (mg/dL) (F), cholesterol (mg/dL) (G), HDL (mg/dL) (H), and LDL (mg/dL) (I) were measured at baseline and up to 15 m post-orchiectomy. HOMA-IR was calculated as (fasting glucose X fasting insulin)/405 (D). Data are expressed as mean ± SEM. ^*^p < .05 by repeated measures one-way ANOVA with Tukey’s multiple comparisons test.

Measuring cortisol in hair can help determine chronic stress levels^30^ and has been utilized in NHP.^31^ Hair cortisol concentration (HCC) has been measured post castration in horses^32^ and bull calves^33^ and shown to be elevated. However, both of these experiments measured acute timepoints within 1 m of castration rather than the further extended postsurgery timepoints in our analysis. Our results demonstrate a significant decrease in HCC at 3 m post-ORX (*p* < .0001) that remains depressed at 15 m post (Figure 6A). Additional circulating measurements of metabolic health were monitored over the time course, which is standard for all animals maintained on WSD. Fasting blood glucose (FBG) significantly increased 3 m post-ORX (*p* = .0154) and remained elevated throughout (Figure 6B). However, there were no significant changes in fasting blood insulin, HOMA-IR, or HbA1c levels in the group (Figure 6C-E). Animals’ lipid levels were also monitored with no change detected in triglycerides but a significant increase in total cholesterol (*p* < .0001), HDL (*p* < .0001), and LDL (*p* = .0437) were all observed by 3 m post-ORX (Figure 6F-I). Interestingly, none of the significant increases in any of the metabolic markers correlated with increased fat mass; although there was a trend with cholesterol (*p*-value = .0661; data not shown).

As part of our routine health maintenance and monitoring, CBC were obtained. Animals demonstrated changes in some parameters of the CBC. Post-ORX animals had a decrease in both neutrophils and monocytes that remained depressed at 15 m post (*p* = .0010 and *p* = .0008, respectively). They also showed a significant increase in lymphocytes (3 m post *p* = .0006, 9 m post *p* = .0399). However, those levels returned to baseline by 15 m postsurgery (Figure S1).

## Discussion

In this study, we examined the impact of ORX on body composition and bone in a colony of DIO-CM. DIO-CM had a wide range of testosterone levels at baseline and higher levels of testosterone did not correlate with greater BMC or BMD at baseline. As expected, both testosterone and inhibin B dropped post-ORX and remained undetectable throughout the 15 m follow-up period. Without testosterone as a substrate to synthesize estradiol, estrogen levels also dropped to undetectable levels and remained so throughout the observation period. The loss of negative feedback when testosterone and inhibin B fell, resulted in large and sustained increases in LH and FSH. Patterns of increase in both LH and FSH post-ORX have been examined in rats, sheep and man. Rats have elevated serum LH and FSH 1-d post-ORX and continue to rise until a peak at 21 d post-ORX.^34^^,^^35^ In sheep, FSH and LH start to rise around 5 d, peak about 14 d, and plateau at an elevated level through 58 d post-castration.^36^ In humans, castration for nondisease purposes, demonstrated maximal levels of FSH within 1-2 wk and LH within 4 wk post-castration which remained elevated compared to intact age-matched men.^37^ The DIO-CM had elevated LH and FSH at 1-wk post-ORX which peaked at 3 m post-ORX (Figure 2). However, a limitation of our study was frequency of blood samples for accurate determination of peak values, without which we are unable to accurately confirm whether the peak in DIO-CM appears delayed vs human or may match the rat model. Overall, this indicates that the hypothalamic-pituitary-gonadal feedback loop may differ between rodents, nonhuman primates, and humans and warrants further studies.

Surprisingly, a decrease in body weight was observed (Figure 5). In these studies, animals had ad libitum access to a diet high in calories (WSD) and just like in domestic pets,^25^^,^^26^ minipigs,^27^ and humans,^28^^,^^29^ we anticipated a significant weight gain post-ORX. Additionally, preexisting obesity prior to initiation of ADT in cancer patients results in the highest risk of greater weight gain posttreatment initiation,^38^ but in contrast, our DIO-CM actually lost weight, roughly 15% on average. However, as expected based on human data, a significant lean mass loss was detected, leading to an overall body fat percentage increase. Additionally, despite the weight loss, our DIO-CM gained trunk fat within 3 m postsurgery (+4.11%) that continued to increase and reached a peak increase of +13% by 15 m post-ORX (Figure 5E). This indicates that our DIO-CM demonstrate similar body composition changes as men treated with ADT who typically loose muscle mass and gain visceral fat mass.^39^ With the concurrent increases in FBG and total cholesterol (mostly due to increases in HDL),^40^ it appears that our NHP model may also be useful to study metabolic syndrome which is present in more than 50% of men undergoing long-term ADT.^28^ Metabolic syndrome is defined as a group of conditions that can consist of increased abdominal obesity, high blood sugar levels and high blood triglycerides/LDL cholesterol, all present in our colony of DIO-CM post ORX.

A major result of this study was the observation that both total body BMC and BMD had a sharp and rapid decline within the first three months post-ORX (Figure 3) accompanied by a massive spike in BTMs prior to hints of stabilization later in the time course (Figure 4). This defines a critical window of time for treatment intervention and bone loss prevention immediately post-ORX. This mirrors the human patient experience where, cancer treatment-induced bone loss is most rapid during the first year of ADT with ~2%-5% BMD loss^41^ and some reports reaching 10% BMD loss.^42^ Acute weight loss can be a significant risk factor for decreased BMD^43^ and could be partially contributing to the bone loss in our DIO-CM post-ORX. However, in previous experiments by our group, loss of BMC/BMD was not observed in rhesus macaques that decreased their body weight by nearly 18% over 12 wk in response to FGF21.^44^ Additionally, rhesus macaques subjected to long term caloric restriction tended to demonstrate lower bone mass then age/sex matched animals on a calorie-replete diet. However, the long term caloric restriction macaques had smaller body frames overall indicating that reduced bone mass is not due to a pathological condition.^45^ Therefore, we expect the major contribution to BMC/BMD loss is ORX and its downstream effects and not weight loss per se.

The majority of older men receiving ADT have either osteopenia or osteoporosis^46^ and many prostate cancer patients have pre-existing low BMD before starting ADT.^47^ Clinical practice recommendations from National Comprehensive Cancer Network (NCCN) Clinical Practice Guidelines in Oncology for bone-heath management in all patients receiving ADT include annual assessment of fracture risk using the Fracture Risk Assessment Tool (FRAX) and supplementation with calcium and vitamin D.^48^ Patients with increased risk via FRAX should also have a baseline DXA with repeated scans every 1-2 yr. If treatment-related bone loss is observed via DXA then pharmacologic therapy with antiresorptive agents (bisphosphonates or denosumab) may be prescribed.^49^ However, there is poor adherence to these guidelines^50^ indicating a missed opportunity at potentially increasing quality of life for these prostate cancer patients. It is important to highlight that castration is different from ADT. However, the negative impact on bone is present in both^51^ indicating that ORX-NHP are a good model to study bone post-sex steroid loss, in addition to studying the effects of new therapies.

Additionally, the disproportionate regional pelvis bone loss demonstrated in our animals replicates the osteoporotic condition in both post-menopausal women and post-ADT men. Morote et al., demonstrated large bone loss in total hip that was most drastic in Ward’s triangle in the neck of the femur.^52^ Unfortunately, our animals only received total body scans and not hip specific scans that would have potentially allowed more finely tuned measurements of specific regions, such as the neck of the femur. Interestingly, it appears that the L Spine as well as the chest cavity (ribs/clavicle/scapula, not vertebrae) were both protected from bone loss. Osteoporosis of the spine most frequently affects the thoracic spine and the thoracolumbar junction, where fractures are most commonly observed due to increased biomechanical stress.^53^^,^^54^ Perhaps this explains protection of the L Spine region specifically; however, this spinal region differs in posture, shape and number between macaques and humans^55^^,^^56^ so this result may not be comparable to human patients and rather a macaque specific phenomenon. The clavicle is the final location of skeletal fusion in humans.^57^ Therefore, it is possible that some animals had not obtained full growth plate fusion at this site and continued to accumulate new bone formation in spite of ORX.

Kessler et al. examined the skeletons of 5 castrated male rhesus macaques and noted that their skulls were smaller, more rounded, had thinner cortical bone and decreased calvarial thickness^18^ compared to intact males. However, 4 of these 5 males were castrated prior to sexual maturity. This is not a finding that has been reported in either human prostate cancer patients on ADT or rat models of ORX. However, cranial sutures, which are the fibrous joints connecting the skull bones, are very different between rats and other primates. Rat cranial sutures remain open throughout adult life and their architecture differs greatly from that of other primates.^58^ Our DIO-CM demonstrated large decreases in both head BMC and BMD that followed the same slope as total body losses, despite post-pubertal status of all males at the time of ORX surgery (Figure 3). Perhaps this has not been reported in humans for a variety of reasons—the head may not be an area of focus during DXA analysis or due to the majority of prostate cancer diagnoses later in life these changes may be obscured due to craniofacial changes with aging.^59^

In males, androgens are secreted mainly by the testis and precursors in lesser amounts by the adrenal gland. Immuno- or surgical castration may alter adrenal gland function to increase androgen.^60^ Cortisol is a steroid hormone produced by the adrenal glands and regulation could be altered due to castration. In human patients, the ADT drug Abiraterone, a CYP17 inhibitor, is known to reduce cortisol levels^61^; however, in human patients orchiectomy does not significantly change serum cortisol levels up to 3-6 m postsurgery.^62^ DIO-CM, similar to castrated rabbits,^63^ demonstrate drastic decreases in cortisol levels post-ORX. However, most published data utilizes salvia for cortisol measurements as opposed to HCC. The hypothalamic-pituitary-adrenal axis may differ between rabbits, rodents, nonhuman primates, and humans and warrants further studies.

We acknowledge that this study does have limitations. For logistical and budgetary reasons, it was not possible to analyze specific cortical and trabecular bone changes or regional muscle mass and muscle composition changes, both of which could have been informative. Additionally, we did not have an age-matched intact control cohort of males as for clinical/behavioral reasons the entire colony underwent ORX. Studies of alterations in bone post-ORX in humans are largely limited to evaluation of patients with a pre-existing cancer diagnosis and frequently do not contain baseline analysis prior to treatment initiation. The ORX rat model allows for longitudinal assessment, but as mentioned previously, a complication to studying bone in the rodent skeleton is that, unlike humans, rodents maintain a growth plate into old age with a potential for longitudinal bone growth much later in their lifespan.^12^ The strengths of this study were utilization of a phenotypically well-characterized experimental group, relevant to human aging as well as muscle and bone biology, to collect longitudinal data pre-ORX and for an extended period post-ORX.

Overall, the study demonstrates that hypogonadism in a colony of DIO-CM induces lean mass loss, increased body fat percentage and numerous hallmarks of osteoporosis recapitulating the key hallmarks of human hypogonadism. This nonhuman primate model could be utilized to test muscle and bone mass preserving interventions in the future, relevant both in age-related hypogonadism and hypogonadism due to medical reasons.

## Supplementary Material

Supplemental_Figure_1_ziag150

## Data Availability

All supporting data will be provided by the corresponding author upon request.
